# IDENTIFYING CONFLICTS EXPERIENCED BY OLDER ADULTS WHILE AGING IN PLACE

**DOI:** 10.1093/geroni/igad104.2636

**Published:** 2023-12-21

**Authors:** Alaine Murawski, Marianne Tschoe, Amber Miller-Winder, Allie Schierer, Raven Relerford, Vanessa Ramirez-Zohfeld, Lee A Lindquist

**Affiliations:** Northwestern University, Chicago, Illinois, United States; Northwestern Medicine, Chicago, Illinois, United States; Northwestern University, Chicago, Illinois, United States; Northwestern University, Chicago, Illinois, United States; Northwestern University, Chicago, Illinois, United States; Northwestern University, Chicago, Illinois, United States; Northwestern University, Chicago, Illinois, United States

## Abstract

Older adults are faced with making decisions about aging-in-place as their health changes. These decisions create conflicts with others causing stress and frustration. We sought to characterize the conflicts older adults experience. As part of a larger study on decision making about aging-in-place (R01AG058777), we are longitudinally following a cohort of older adults with surveys every 6 months. Subjects are asked to describe conflicts that have occurred regarding their living situation. Responses were coded using constant comparative analysis examining type, content, and whether statements were interests, rights, or power-based. We enrolled 293 subjects (mean age 73.5 yrs, SD 5 yrs, 40.4% non-White; 94.5% retention rate) and 124 conflicts were reported over 18 months. Thematic analysis revealed three types of conflicts: Interpersonal (subcategories: spouse, intergeneration, other), task, and value-based. Content was coded into 6 themes: Location change, home maintenance issues (e.g., My son insisted I put in a new lower tub, and I didn’t want to), different ways of completing tasks (e.g., it is about my children and the way we do things), safety (e.g., Neighbor’s boyfriend kicked in the door and destroyed our property), financial, and health-related. Most statements were either interests or rights-based. Older adults experience a range of conflicts involving aging-in-place decision making. Most often older adults experience interpersonal conflicts with supporters about moving from their home or maintaining/adapting their home. Training in negotiation may help alleviate these conflicts between older adults and their care supporters.

